# Trajectories of clinical and parenting outcomes following admission to an inpatient mother-baby unit

**DOI:** 10.1186/s12888-019-2331-0

**Published:** 2019-11-01

**Authors:** Nicole Reilly, Elloise Brake, Nancy Briggs, Marie-Paule Austin

**Affiliations:** 1 Research Centre for Generational Health and Ageing & School of Nursing and Midwifery, Faculty of Health and Medicine, University of Newcastle, HMRI Building Lot 1 Kookaburra Circuit, New Lambton Heights, NSW 2305 Australia; 2 Perinatal and Women’s Mental Health Unit, St John of God Health Care and University of New South Wales, 13 Grantham St, Burwood, 2134 Australia; 3 Stats Central, Mark Wainwright Analytical Centre, Biological Sciences South Building, UNSW, Sydney, 2052 Australia

**Keywords:** Mother-baby unit, Mental health outcomes, Parenting outcomes

## Abstract

**Background:**

This study aimed to examine trajectories of clinical and parenting outcomes following admission to a mother-baby unit (MBU), and to explore factors associated with these trajectories.

**Methods:**

Women admitted to an MBU completed the Edinburgh Postnatal Depression Scale (EPDS), Depression, Anxiety and Stress Scale (DASS-21), Karitane Parenting Confidence Scale (KPCS) and Maternal Postnatal Attachment Scale (MPAS) at admission, discharge and 3 months following discharge. Questions assessing psychosocial risk and adult attachment style were also completed at admission, and information relating to service engagement in the time since discharge was collected at follow-up. Additional clinical and demographic information was extracted from the patient medical record.

**Results:**

Seventy-five women participated in the study. Overall, significant improvements in mean scores on measures of anxiety and parenting confidence were maintained 3-months following discharge. However, the majority of women (93.3%) followed trajectories that were characterised by deterioration in self-reported mother-infant attachment following discharge. 62.9 and 34.6% of women followed trajectories of increased symptoms of depression and stress between discharge and follow-up, respectively. Across measures, the least optimal trajectories, or least optimal scores, at follow-up were associated with less secure maternal attachment style (associated with more anxiety symptoms, poorer parenting confidence and maternal-infant attachment), older maternal age (more depressive symptoms) and increased psychosocial risk (more anxiety symptoms).

**Conclusions:**

The findings of this study highlight the clinical implications of anxious attachment style for the mental health and parenting outcomes of women admitted to an MBU and the importance of incorporating mother-infant therapy as part of an ongoing management plan. Comprehensive discharge planning and transitional care to help ensure women discharged from an MBU are best supported in the longer term is recommended.

## Introduction

Admission of women experiencing moderate to severe perinatal mental illness to an inpatient mother-baby unit (MBU), where these facilities are available and appropriate, is now considered clinical best practice in a number of developed countries, including Australia and the United Kingdom [1, 2]. These specialist units allow women to be admitted jointly with their infant, and provide expert mental health management as well as support for women in caring for and developing a relationship with their baby.

Recent reviews have shown that admission to an MBU positively impacts on maternal mental health and the mother-infant relationship, with significant improvements across a range of clinical and parenting outcome measures from admission to discharge [3–5]. However, only six studies have included a post-discharge follow-up of women admitted to an MBU, to assess lasting change [3]. Bardon and colleagues, for example, reported that 48% of women followed up nearly 3 years after discharge had a poor or moderate ‘state of adjustment’, as subjectively assessed by interview, doctor report or self-report [6]. More recently, a follow-up study of 55 women admitted to a Belgian MBU with severe postpartum depression found that 39% reported moderate to severe symptoms of depression 3.5 years after discharge, with women with high levels of self-criticism particularly vulnerable to poorer outcomes [7, 8]. High overall rates of recurrence (87.2%) and readmission (63.3%) over a 10-year follow-up period have also been reported [9].

Studies which have examined parenting outcomes following discharge have shown that severe postnatal mental illness continues to negatively impact on mother-infant interactions, with women previously admitted to an MBU observed to be less sensitive, less appropriate and more negative in their play over the first year postpartum compared to a community-based depressed control group [10]. Post-discharge parenting outcomes have been shown to be strongly associated with diagnostic group (with women with psychotic disorders at a particular disadvantage) as well as a woman’s social context [5].

A recent review called for more follow-up studies of women discharged from an MBU that include a focus on attachment and parenting [3]. The current study responds to this call by examining trajectories of clinical and parenting outcomes following admission to an Australian MBU, and exploring factors associated with these trajectories.

## Materials and methods

### Setting

This study recruited women admitted to a private MBU in Sydney, Australia, between 3 August 2012 and 17 October 2016. This 12-bed purpose-built unit provides multidisciplinary care to mothers experiencing postnatal depression, anxiety and other related conditions, as well as to their babies and their partners. The inpatient group program includes skills based groups underpinned by dialectical behaviour therapy and cognitive behaviour therapy principles, attachment-based groups, anxiety management, mindfulness, mothercraft groups, art and music therapy. All admissions to this MBU are voluntary, and require private health insurance and referral from a health care professional. The most severely ill mothers needing involuntary admission are cared for in the public health system. This MBU is the only inpatient service of its type in NSW, Australia’s most populous state, and referrals to the service are received from a vast geographic area.

The research study was approved by the institutional Human Research Ethics Committee (Ref. # 402). Women who agreed to participate gave written consent and were asked to complete computer-based self-report measures shortly after admission, at the time of discharge and approximately 15 weeks post-discharge. Participants also consented to their inpatient information being used for research purposes.

### Measures

Data for this analysis were collected using the following self-report questionnaires:

*The Edinburgh Postnatal Depression Scale (EPDS)* [11]: a 10-item self-report scale designed as a screening instrument for perinatal depression. Respondents rate the intensity of depressive symptoms present within the previous 7 days, and a score of 13 or more has been shown to have moderate sensitivity (0.67–1.00) and high specificity (consistently 0.87 or above) for detecting possible major depression in postpartum women [12].

*Depression, Anxiety and Stress Scale − 21 (DASS-21)* [13]: a 21-item short form of the Depression Anxiety Stress Scales. The DASS-21 has three independent scales, each comprising seven items representing the dimensions of depression, anxiety and stress. Respondents are asked to rate the extent to which they experienced each state over the past week, and a series of cut-off values can be used to classify individuals into severity rating categories [13]. Only the anxiety and stress subscales were included in analyses for the current study.

*Karitane Parenting Confidence Scale (KPCS)* [14]: a 15-item self-report questionnaire assessing perceived parenting confidence. Items are rated on a 4 point Likert scale, with lower scores indicating lower parenting self-efficacy. Although the clinical cut-off score for the KPCS is 39 or less, it has been suggested that focusing on scores within the moderate and severe clinical range (35 or less) may be more practical in clinical and evaluation settings [15].

*Maternal Postnatal Attachment Scale (MPAS)* [16]: a 19-item self-report questionnaire assessing the domains of pleasure in proximity, acceptance, tolerance and competence as a parent. A higher score indicates more optimal attachment of the mother to her infant. The three subscales of the MPAS are quality of attachment, absence of hostility and pleasure in interaction. No clinically significant threshold has been established, however a mean score of 84.6 has been reported in a community sample of women at 4 months post-partum [16].

*Attachment Style Questionnaire (ASQ)* [17]: a 40-item self-report scale designed to assess attachment style in adults. Participants are asked to rate the extent to which items describe their feelings and behaviour in close relationships using a 6-point scale. The ASQ has been shown to have both 5-factor and 3-factor solutions, with the 3-factor solution yielding ‘Security’, ‘Anxiety’ and ‘Avoidance’ factors. In line with previous research (e.g. [18], the Anxious and Avoidance subscales of the 3-factor solution were used in the current study.

*Postnatal Risk Questionnaire (PNRQ)* [19, 20]: a 12-item self-report measure developed to assess postnatal women for the presence of psychosocial risk factors known to be associated with the onset of perinatal mental disorders, in particular perinatal depression and anxiety disorders. The PNRQ is based on the validated Antenatal Risk Questionnaire [19], with additional postnatal items relating to birth experience, parenting experience and infant feeding and settling problems. Higher scores on the PNRQ indicate greater psychosocial risk.

The EPDS, DASS-21, KPCS and MPAS were completed at admission, discharge and follow-up. The ASQ and PNRQ were completed at admission only. In addition, information relating to the type and number of engagements with a range of health services and treatment options in the time since discharge was collected as part of the follow-up survey. Additional demographic and clinical information including maternal diagnosis and length of stay were collected from patient files.

### Statistical analysis

Descriptive statistics were used to examine the demographic and psychosocial profiles of women who were admitted to the MBU during the data collection period. Characteristics of women who were and were not included in the current analysis were compared using chi-square tests or t-tests.

Total scores and sub-scale scores for all questionnaires (EPDS, DASS-21, KPCS, MPAS, ASQ, PNRQ) were calculated according to authors’ scoring instructions. Engagement with various health services and providers was operationalised dichotomously (yes/no) and total number of service engagements was calculated as the sum of all engagements since discharge (all service types). Information relating to medication use since discharge was coded as: continued medication; changed/stopped medication; no medication.

Group-based latent class growth modelling [21, 22] was used to identify trajectories of scores on the clinical and parenting outcome measures across three time points (admission, discharge and follow-up) in this sample of women. Censored normal models were fitted by testing linear and quadratic trends for each. The number of classes and order of each class were determined by included likelihood ratio statistics (L), Akaike Information Criterion (AIC) and Bayesian Information Criterion (BIC) (with better fitting models having lower values) and substantive interpretation of the resulting classes. Finally, maternal and infant age, parity, diagnostic group, psychosocial risk (PNRQ score), maternal attachment insecurity (ASQ anxious and avoidant subscale scores) and service engagement since discharge were examined as predictors of latent class membership across each of the outcome measures.

Data were analysed using IBM SPSS Statistics version 24 [23] and SAS 9.4 [24]. Proc Traj was used to estimate the models [21, 25].

## Results

A total of 437 women were admitted to the MBU during the study period. Of these, 378 (86.4%) and 391 (89.5%) women completed the admission and discharge measures, respectively. At the time of discharge, 215 women (55.0%) indicated their willingness to be contacted by the research team 3-months later. Online follow-up surveys were subsequently completed by 96 eligible women (44.7%). Of those that completed the follow-up survey, 84 (87.5%) had complete admission, discharge and 3-month data. Of these, nine women were excluded from further analysis as they had not consented to their inpatient information being used for research (rather than quality improvement) purposes. The final dataset thus comprised 75 women. The mean length of time between discharge and completion of the follow-up measures was 15.13 weeks (SD = 4.19; range 11–37 weeks) and mean infant age at follow up was 6.6 months, with 61.3% first borns. Three-quarters of women (76%) had a primary diagnosis of a mood disorder (primarily major depressive episode), 16% an anxiety disorder, and 8% puerperal psychosis. Forty-nine women (65%) fulfilled criteria for at least one comorbid psychiatric diagnosis; of these, 66% had a comorbid depressive or anxiety disorder. Participants’ demographic, psychosocial and clinical characteristics are presented in Table 1. There were no significant differences between women admitted to the MBU during the data collection period who were and were not included in the current follow-up analysis in terms of primary diagnostic group, maternal age, infant age, infant gender, length of stay, parity, partner status, country of birth, PNRQ or EPDS scores at admission, or DASS-21, KPCS, MPAS scores at admission or discharge. Women who participated in the follow-up had a lower EPDS score at discharge (M = 8.83, SD = 4.11) than women who did not (M = 9.98, SD = 5.23; t (310) = − 1.98, *p* = .049), however the magnitude of this difference was small (eta squared = .012). A summary of participants’ engagement with support services in the time since discharge is provided in Table 2.

**Table 1:** Participant characteristics (*n* = 75)

		N (%)
Maternal age	M = 33.80 (SD = 4.10; range 24–48 years)	
	< 25 yrs	1 (1.3)
	26 – 35 yrs	51 (68)
	36 – 49 yrs	23 (30.7)
Partner status	Married/ De facto	73 (97.3)
	Separated/ Divorced/	1 (1.30)
	Single	1 (1.30)
Parity	One	46 (61.3)
	Two	21 (28)
	Three	8 (10.7)
Country of birth	Australia	62 (82.7)
	Other	13 (17.3)
Length of stay (days)	M = 25.35 (SD = 12.45)	
Primary diagnosis (this admission)	Mood disorder^a^	57 (76.0)
	Anxiety disorder^b^	12 (16.0)
	Puerperal psychosis	6 (8.0)
Infant age at admission (months)	M = 3.49 (SD = 3.47)	
	0–3 months	49 (65.3)
	4–6 months	8 (10.7)
	7–9 months	13 (17.3)
	10–15 months	5 (6.7)
Infant age at follow-up (months)	M = 6.59 (SD = 3.73)	
Infant gender	Male	44 (58.6%)
	Female	29 (38.7%)
	Female – Male Twins	2 (2.7%)

^a^ Includes *n* = 55 Depressive Disorder, n = 2 Bipolar Disorder Mixed; ^b^ Includes n = 8 Generalised Anxiety Disorder, *n* = 2 Panic Disorder, *n* = 1 Obsessive Compulsive Disorder, *n* = 1 Post Traumatic Stress Disorder

**Table 2:** Participant engagement with mental health treatment and support services since discharge (*n* = 75)

	Total *N* = 75 N [%]	Number of engagements, by service group M (SD)
Any mental health treatment or support^a^	64 [85.3]	–
General Practitioner	40 [53.3]	3.2 [2.8]
Mental Health Nurse	10 [13.3]	6.9 [4.5]
Psychologist	43 [57.3]	6.3 [3.1]
Psychiatrist	54 [72.0]	5.3 [5.1]
Therapy groups	26 [34.7]	6.0 [3.3]
Medication – ever	58 [77.3]	–
Medication – continued	39 [52.0]	–
Medication – changed	9 [12.0]	–
Medication – stopped	10 [13.3]	–

^a^That is, engaged with at least one of the treatment or support options listed. Participants may have engaged with more than one type of treatment or support — hence % totals more than 100%

### Trajectories of scores on clinical and parenting outcome measures across admission, discharge and follow-up

Descriptive statistics for the EPDS, DASS-21, KPCS and MPAS at admission, discharge and follow-up, and the proportions of women scoring in the clinical range at each time point, are presented in Additional file 1: Table S1. The final model fit indices for latent classes of scores on these measures from admission to follow-up are presented in Additional file 2: Table S2. Descriptive statistics by group classification for each final model are detailed in Additional file 3: Table S3.

#### Depressive symptom trajectories (EPDS)

**Fig. 1:**
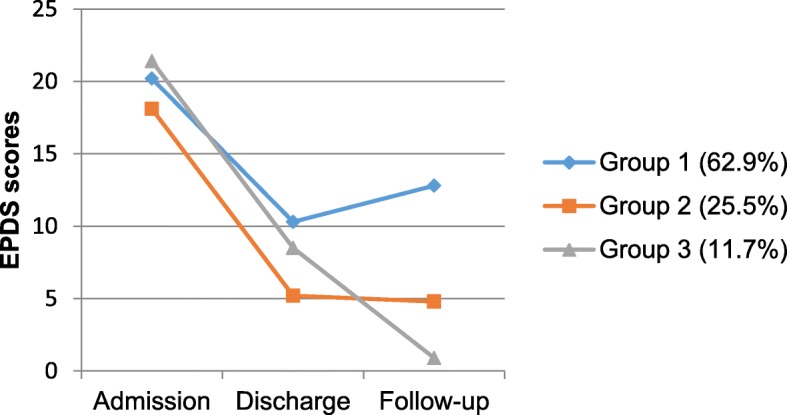
Estimated means of the EPDS for three trajectories of depressive symptoms from admission to follow-up

The final model showed three trajectories of depressive symptoms from admission to follow-up, with 62.9% (*n* = 48), 25.5% (*n* = 19) and 11.7% (*n* = 8) women classified in EPDS group 1, group 2 and group 3, respectively (see Fig. 1). Each group showed a decrease in EPDS scores from admission to discharge (effect sizes of change ranging from *d* = 1.84 in group 1 to *d* = 3.11 in group 2), with over a third of women maintaining these improvements (group 2, *d* = 0.07) or further improving (group 3, *d* = 3.00) after returning home. However, the mean EPDS score for the nearly two-thirds of women in group 1 was slightly higher at follow-up than at discharge (M = 12.8 vs M = 10.8, *d* = 0.37), with 41.7% of group 1 women also scoring at or above the clinical range of 13 or more at follow-up.

#### Anxiety symptom trajectories (DASS-21-anxiety)

**Fig. 2:**
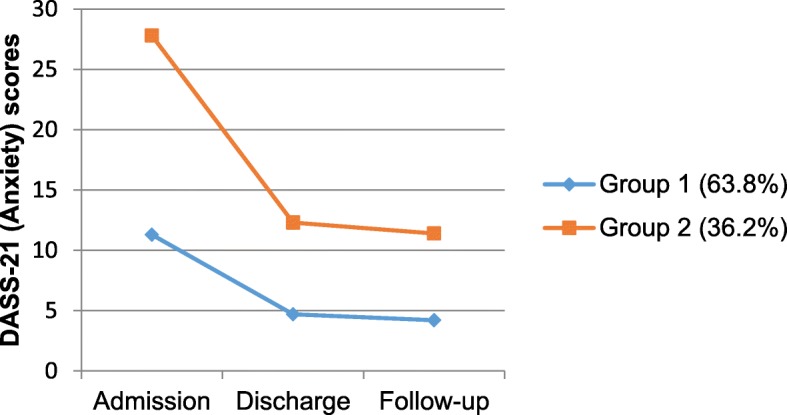
Estimated means of the DASS-21 (Anxiety) for two trajectories of anxiety symptoms from admission to follow-up

Two distinct trajectories of anxiety symptoms from admission to follow-up were identified in the final model, with 54 women (63.8%) classified in Anxiety group 1 and 21 women (36.2%) classified in Anxiety group 2. Both groups showed a decrease in anxiety from admission to discharge (*d =* 0.83 and *d =* 1.81, respectively) and maintenance of these improvements after returning home from the MBU (*d =* 0.09 and *d =* 0.09, respectively). However, women in group 2 had higher anxiety scores at each time point, with these elevated scores remaining in the moderate clinical range at follow-up (see Fig. 2).

#### Stress symptom trajectories (DASS-21-stress)

**Fig. 3:**
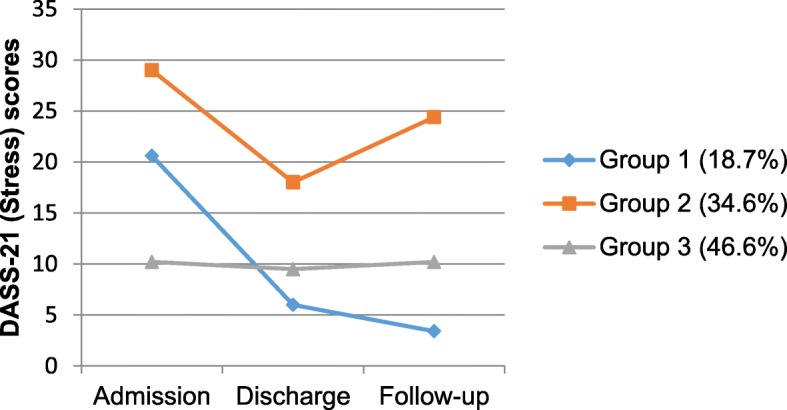
Estimated means of the DASS-21 (Stress) for three trajectories of stress symptoms from admission to follow-up

As for EPDS scores, the final model for scores on the DASS-21 Stress subscale showed three distinct trajectories of stress symptoms from admission to follow-up, with 18.7% (*n* = 13), 34.6% (*n* = 27) and 46.6% (*n* = 35) women classified in Stress group 1, group 2 and group 3, respectively (see Fig. 3). Each group showed a decrease in DASS-Stress scores from admission to discharge (*d =* 3.06, *d =* 1.19 and *d =* 2.20, respectively), with two-thirds of women maintaining these improvements (group 3, *d =* 0.24) or further improving (group 1, *d =* 1.17) after returning home. Women in group 2 had higher stress scores at follow-up than at discharge (M = 24.4 vs M = 18.0, *d =* 0.65), with 70.4% of these women also scoring at or above the moderate clinical range.

#### Parenting confidence trajectories (KPCS)

**Fig. 4:**
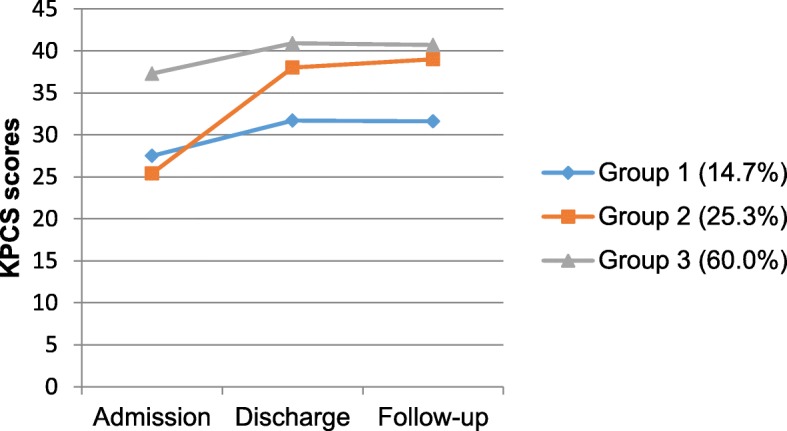
Estimated means of the KPCS for three trajectories of parenting confidence from admission to follow-up

Three distinct trajectories of scores on the KPCS were identified, with the smallest group of women (*n* = 11; 14.7%) scoring in the moderate to severe clinical range for parenting confidence at each time point. Women in the second trajectory (*n* = 19; 25.3%) showed the most improvement in KPCS scores over the course of their MBU admission (mean scores 25.4 vs 38.0, *d =* 5.07, indicating a shift from the severe to mild clinical range), with these improvements maintained at the 3-month follow-up (*d =* 0.28). The third and largest group (*n* = 45; 60.0%) had the highest parenting confidence scores at each time point, with scores remaining in the non-clinical range at discharge and after returning home (see Fig. 4).

#### Maternal attachment trajectories (MPAS)

**Fig. 5:**
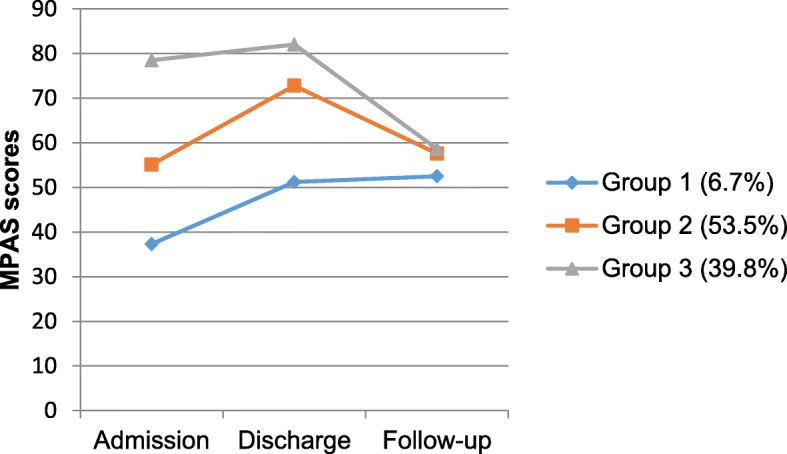
Estimated means of the MPAS for three trajectories of maternal attachment from admission to follow-up

The final model for maternal attachment showed three trajectories of scores over time. The smallest group (*n* = 5; 6.7%) had the lowest mean scores at each time point, however for these women, improvements in MPAS scores gained between admission and discharge (mean scores 37.3 vs 51.2, *d =* 1.12) were maintained at follow-up (*d =* 0.13). Women in group 2 (*n* = 40; 53.5%) showed the greatest improvement in self-reported maternal attachment over the course of the admission (mean scores 55.1 vs 72.8, *d =* 1.79). However, MPAS scores deteriorated markedly after discharge for women in both group 2 (*d = 1.82)* and group 3 (*d =* 3.54), with mean scores approaching the group 1 mean at follow-up (see Fig. 5).

### Factors associated with trajectories of scores on clinical and parenting outcome measures

Anxious maternal attachment style, as measured by the ASQ Anxious subscale, was a significant predictor of latent trajectories for anxiety symptoms, parenting confidence and maternal-infant attachment. Specifically, women with a more anxious attachment style were more likely to follow a course of elevated DASS-21 anxiety scores (β = 1.4, *p* = 0.038), and lowest – or least optimal – scores on the KPCS (β = − 1.38, *p* = 0.01) and MPAS (β = − 2.11, *p* < 0.01), from admission to follow-up. Women with a more avoidant attachment style were also more likely to follow a trajectory of lower parenting confidence scores, with mean scores remaining in the moderate-severe clinical range across admission, discharge and follow-up (β = − 1.17, *p* < 0.03).

Maternal age was not a significant predictor of trajectory classification across DASS-21 (anxiety and stress), KPCS or MPAS scores (*p* values >.05). However women who were older were *less* likely to have maintained improvements, or further improved, in terms of scores on the EPDS after discharge (β = − 0.25, *p* = 0.02, and β = − 0.25, *p* = 0.05, respectively). Psychosocial risk was a significant predictor of latent trajectories for anxiety symptoms only. Specifically, a one unit increase in the total psychosocial (PNRQ) score was associated with higher odds of following a course of elevated DASS-21 anxiety scores from admission to follow-up (β = 0.06, *p* = 0.04).

There was no significant association between infant age, parity, diagnostic group or level of service engagement since discharge and trajectory group membership for any of the clinical or parenting outcome measures.

## Discussion

This study of 75 women admitted to an Australian MBU showed that overall, significant improvements in mean scores on measures of anxiety and parenting confidence were maintained 3-months following discharge. However, closer inspection of women’s scores showed distinct, and in some cases suboptimal, trajectories of clinical and parenting outcomes over time, despite many women reporting frequent engagement with mental health services post-discharge. The majority of women followed a trajectory that was characterised by a decline in self-reported mother-infant attachment following discharge, and a significant proportion of women followed trajectories of increased symptoms of depression and stress at follow-up. Anxiety symptoms for all women improved during the admission and remained stable at follow-up, however scores remained in the moderate clinical range for over a third of women. Across measures, the least optimal trajectories, or least optimal scores, at follow-up were associated with less secure maternal attachment style (associated with more anxiety symptoms, poorer parenting confidence and maternal-infant attachment), greater maternal age (more depressive symptoms) and increased psychosocial risk (more anxiety symptoms).

Deterioration in maternal-infant attachment from discharge to follow-up – which was observed in over 90% of women – was perhaps the most striking finding to emerge from this study. A recent qualitative study has also described the difficulties women can experience building and maintaining a relationship with their child following admission to an MBU, as well as guilt about how their emotional state negatively impacts on their ability to parent their child [26]. Although most MBUs include a specific focus on the mother-infant relationship as part of their inpatient treatment program [3], a recent review concluded that there is only a low certainty of evidence to show that mother-infant relationship interventions improve mother-infant attachment problems among women with postnatal depression, with any benefits of these interventions limited to the immediate term and to individual rather than groups-based programs [27]. While an MBU admission provides a unique opportunity to engage mothers in conversations around mother-infant attachment and to increase her confidence in the mothering role, the treatment of more complex presentations and parenting difficulties should be acknowledged as outside the scope of these more acute, short-term inpatient services. This is particularly the case in countries including Australia, where there is an increasing shift towards community-based mental health services rather than extended episodes of inpatient care, and where the average length of an MBU admission is 22 days [28–30].

The findings of this study highlight the importance of incorporating mother-infant therapy from the outset of the admission (where appropriate), as part of a recovery based early discharge plan. However, additional studies that examine the benefit of community-based (individual and group) mother-infant interventions are clearly needed. A recent evaluation of the 20-week Circle of Security group-based program has shown that this attachment-based intervention is associated with significant improvements in the emotional functioning of caregivers of young children (aged 1–7 years), particularly those who report high levels of stress at the beginning of the program [31], as well as significant caregiver-child relationship improvements [32]. Similarly, a randomised-controlled trial of the attachment-based 13-week Mom Power program (comprising three weekly individual sessions and 10 weekly group sessions) also demonstrated enhanced reflective capacity and improved maternal representations of their child (mean age 15 months) among a sample of high risk women [33].

While clinical improvements gained during the admission were maintained in terms of anxiety symptoms, nearly two-thirds of women in this study followed a trajectory of higher EPDS scores at follow-up than at discharge. Although the mean difference in scores for women in this group was small (M = 10.8 at discharge vs 12.8 at follow-up), over 40% of these women scored in the clinical range after returning home, which is consistent with previous studies of this type (e.g., [7, 8]). In addition, self-reported stress symptoms were greater at follow-up than at discharge for nearly 35% of women. While none of the predictors entered into the model were found to be associated with the observed patterns of stress scores, previous studies have described the significant increases in parenting, household and occupational responsibilities that women face after returning home, and how these can be experienced as overwhelming [26, 30]. Although these issues were not specifically explored in this sample, it is reasonable to assume that as in other studies, some women would have found the transition from the MBU environment, where intensive nursing, mothercraft and specialist support was readily available, to the home environment immensely challenging. Engagement with community-based in-home support packages is one strategy that may help make the transition home less confronting for women and their families, however a clear understanding of the role of the in-home support team, timeframes for care and a gradual reduction in the level of practical support provided will be necessary to circumvent any unintended dependence on these services in the longer term.

This study also showed that an anxious attachment style, as measured by the ASQ, was associated with less optimal parenting confidence and mother-infant attachment trajectories, as well as consistently elevated anxiety scores. These findings are in line with previous work conducted in other clinical and community-based samples. For example, an earlier study of 83 women admitted to an Australian residential parenting facility showed that anxious and avoidant attachment predisposes women to parenting self-efficacy difficulties, particularly in the presence of maternal depression [18]. An insecure attachment style has also been found to be predictive of both antenatal and postnatal mood disturbance [34], with a more recent study reporting that maternal depression, presenting in combination with a disorganised attachment style, poses a particular risk for the developing mother-infant bond [35]. Taken together, these studies highlight the importance of routinely assessing the attachment and personality factors which may influence short- and longer-term outcomes for mothers and their babies from the outset of the admission. An important implication of the current study is that women with an anxious attachment style are particularly vulnerable to mental health and parenting issues following the birth of an infant. Assessment of this before birth could potentially help women and their families, if these women were targeted for extra support.

The time to follow-up in the current study was relatively brief compared to other studies that have examined post-discharge outcomes for women admitted to an MBU (e.g., [6–9]). Yet our results clearly demonstrate that deterioration of clinical symptoms and resumption of mother-infant attachment difficulties can occur even in these short intervening periods. These outcomes were observed despite a high proportion of women in the sample remaining on medication and reporting frequent engagement with mental health services during the time since discharge. While we are unable to comment more fully on the treatment focus of the post-discharge care that was received by women, these findings align with previous work which has shown that ongoing antidepressant treatment, for example, does not improve maternal-infant interaction in women with postpartum depression [36].

The importance of structured discharge plans that are well-understood not only by women, but also their carers and their health care providers, cannot be understated. While we did not ask women specifically about their personal commitment to their own help seeking behaviour following discharge, previous research suggests that an impaired ability to make decisions after returning home from an MBU can contribute to passive acceptance and attendance at appointments organised for them [26]. Ideally, comprehensive discharge planning and transitional care should begin from the time of the admission to help ensure that the management and treatment of what are often complex presentations are individualised and best supported in the longer term.

Several limitations of this study must be acknowledged. Trajectories for each clinical and parenting outcome were reported separately due to the small sample size and bootstrapping techniques to examine the stability of subgroups were not applied. Larger studies that profile overall trajectories by including all outcomes in a single latent class analysis are warranted. Additional limitations include the absence of information about the quality of intimate partner relationships and short interval to post-discharge follow-up, The sample had a high proportion of women with depressive or anxiety disorders and participants are assumed to be of mid-high socioeconomic status, given top tier private health insurance is a requirement for admission to the MBU. This limits the generalisability of our results to women with more severe, low prevalence diagnoses and to women admitted to publicly funded units.

The use of self-report measures to assess clinical and parenting outcomes of interest may also be considered problematic due to the potential for reporter-bias. For example, past studies have shown that women with postnatal depression self-report a significantly more negative experience of bond with their child than women with postpartum psychosis [37]. However the inclusion of repeated observer-rated mother-infant interaction assessments rather than self-reported mother-infant attachment, or the Adult Attachment Interview (AAI) rather than the ASQ, for example, were beyond the resource limits of this study. In addition, the clinical appropriateness of administering the AAI in this setting is questionable, given the acute vulnerability and diminished mental state of women, particularly at the beginning of their stay on the ward. Past research has shown that borderline personality disorder and borderline personality traits are prevalent in MBU populations [29].

## Conclusions

This study responds to calls for more evidence relating to mental health outcomes for women who have been admitted to an MBU [38]. To our knowledge, it is also the first study to use latent class modelling to examine the trajectories of clinical and parenting outcomes. Findings highlight the clinical implications of anxious attachment style for the mental health and parenting outcomes of women admitted to an MBU and the importance of incorporating mother-infant therapy as part of a comprehensive discharge and transitional care plan to help ensure that women and families are best supported in the longer term. Future studies which explore the impact of personality vulnerabilities, including emotional dysregulation, on short and longer term outcomes for women admitted to an MBU may enhance the provision of tailored therapeutic interventions to further improve the outcomes of at risk mother-infant dyads. Additional qualitative studies which more fully articulate a woman’s lived experience following discharge from an MBU would be of great value to clinicians working with mothers and their families at this critical time. The potential impact of geographical disparities in access to specialised treatment and support for these particularly vulnerable women and their families also warrants further investigation. Finally, given the increasing shift towards recovery-oriented mental health services [39], it will be important for future research to examine the role of the MBU in nurturing hope, optimism and empowerment alongside improvements in clinical symptoms and parenting capacity.

## Supplementary information

**Additional file 1: Table S1.** Descriptive statistics for clinical and parenting outcome measures at admission, discharge and follow-up (*n* = 75).

**Additional file 2: Table S2.** Final model fit for latent classes of scores on clinical and parenting outcome measures at admission, discharge and follow-up (*n* = 75).

**Additional file 3: Table S3.** Descriptive statistics for clinical and parenting outcome measures by group classification, for each final model (*n* = 75).

## Data Availability

The dataset generated and analysed in the current study cannot be released to third parties without prior HREC approval.

## Abbreviations

AAI: Adult Attachment Interview
ASQ: Attachment Style Questionnaire
DASS-21: Depression, Anxiety and Stress Scale (21-item version)
EPDS: Edinburgh Postnatal Depression Scale (EPDS)
KPCS: Karitane Parenting Confidence Scale
MBU: Mother-baby unit
MPAS: Maternal Postnatal Attachment Scale
PNRQ: Postnatal Risk Questionnaire
