# Supplementary material for: Trajectories of clinical and parenting outcomes following admission to an inpatient mother-baby unit
Source: BMC Psychiatry. 2019 Nov 1;19:336. doi: 10.1186/s12888-019-2331-0 (PMC6825337; doi:10.1186/s12888-019-2331-0)
Supplement: Supplementary file 1 — Additional file 1: Table S1. Descriptive statistics for clinical and parenting outcome measures at admission, discharge and follow-up (n = 75). [file 12888_2019_2331_MOESM1_ESM.docx]

**Supplementary Table 1. Descriptive statistics for clinical and parenting outcome measures at admission, discharge and follow-up (n=75)**

|  | **M (SD)** | | | **Proportion above clinical cut-point**^$^  **N (%)** | | |
| --- | --- | --- | --- | --- | --- | --- |
| **Measure** | **Admission** | **Discharge** | **Follow-up** | **Admission** | **Discharge** | **Follow-up** |
| EPDS | 19.80 (4.01) | 8.82 (4.11) | 9.49 (6.52) | 72 (96.0) | 13 (17.3) | 20 (26.7) |
| DASS-21 Anxiety | 16.59 (10.08) | 7.15 (6.39) | 6.51 (7.51) | 55 (73.3) | 26 (34.7) | 21 (28.0) |
| DASS-21 Stress | 27.47 (8.14) | 11.97 (6.77) | 14.12 (9.86) | 62 (82.7) | 12 (16.0) | 20 (26.7) |
| KPCS | 32.85 (6.66) | 38.83 (3.84) | 38.89 (4.42) | 44 (58.7) | 14 (18.7) | 13 (17.3) |
| MPAS Total | 63.29 (14.80) | 75.03 (12.03) | 57.66 (4.89) | 59 (78.7) | 42 (56.0) | 75 (100.0) |
| *Quality of Attachment* | *30.80 (7.89)* | *38.21 (4.69)* | *29.81 (3.35)* | *-* | *-* | *-* |
| *Absence of Hostility* | *15.26 (4.12)* | *17.29 (3.32)* | *17.49 (3.84)* | *-* | *-* | *-* |
| *Pleasure in Interaction* | *17.43 (4.85)* | *19.85 (3.69)* | *10.36 (4.24)* | *-* | *-* | *-* |

EPDS: Edinburgh Postnatal Depression Scale; DASS-21: Depression, Anxiety and Stress Scale-21 item; KPCS: Karitane Parenting Confidence Scale; MPAS: Maternal Postnatal Attachment Scale

^$^ Clinical cut-points: EPDS≥13; DASS-21-Anxiety≥10 (severity rating of moderate or above); DASS-21-Stress≥19 (severity rating of moderate or above); KPCS≤35 (moderate-severe clinical range); MPAS≤77 (calculated as ≥1 SD below community mean score (Condon and Corkindale, 1998).
