# Supplementary material for: Trajectories of clinical and parenting outcomes following admission to an inpatient mother-baby unit
Source: BMC Psychiatry. 2019 Nov 1;19:336. doi: 10.1186/s12888-019-2331-0 (PMC6825337; doi:10.1186/s12888-019-2331-0)
Supplement: Supplementary file 2 — Additional file 2: Table S2. Final model fit for latent classes of scores on clinical and parenting outcome measures at admission, discharge and follow-up (n = 75). [file 12888_2019_2331_MOESM2_ESM.docx]

**Supplementary Table 2. Final model fit for latent classes of scores on clinical and parenting outcome measures at admission, discharge and follow-up (n=75)**

| **Measure** | **Number of classes (order)** | **L** | **BIC** | **AIC** | **p-value** |
| --- | --- | --- | --- | --- | --- |
| **EPDS** | 3-class (2,2,2) | -141.23 | -167.13 | -153.23 | <.001 |
| **DASS-21 Anxiety** | 2-class (2,2) | -707.21 | -724.48 | -715.21 | <.001 |
| **DASS-21 Stress** | 3-class (2,2,2) | -136.83 | -162.74 | -148.83 | <.001 |
| **KPCS** | 3-class (1,2,2) | -636.93 | -660.68 | -647.93 | .001 |
| **MPAS Total** | 3-class (1,2,2) | -783.72 | -807.47 | -794.72 | <.001 |

EPDS: Edinburgh Postnatal Depression Scale; DASS-21: Depression, Anxiety and Stress Scale-21 item; KPCS: Karitane Parenting Confidence Scale; MPAS: Maternal Postnatal Attachment Scale; L: Likelihood ratio statistics; AIC: Akaike Information Criterion; BIC: Bayesian Information Criterion
