# Supplementary material for: Trajectories of clinical and parenting outcomes following admission to an inpatient mother-baby unit
Source: BMC Psychiatry. 2019 Nov 1;19:336. doi: 10.1186/s12888-019-2331-0 (PMC6825337; doi:10.1186/s12888-019-2331-0)
Supplement: Supplementary file 3 — Additional file 3: Table S3. Descriptive statistics for clinical and parenting outcome measures by group classification, for each final model (n = 75). [file 12888_2019_2331_MOESM3_ESM.docx]

**Supplementary Table 3. Descriptive statistics for clinical and parenting outcome measures by group classification, for each final model (n=75)**

| **Measure** | **Group** | **N**  **(%)** | **Data collection point** | **Mean** | **SD** | **Minimum** | **50th Pctl** | **Maximum** |
| --- | --- | --- | --- | --- | --- | --- | --- | --- |
| EPDS Total Score | 1 | 48 (62.9) | Admission  Discharge  Follow-up | 20.23 10.33 12.77 | 4.13 3.76 5.71 | 11.00 4.00 6.00 | 20.50 9.50 11.00 | 28.00 19.00 28.00 |
|  | 2 | 19 (25.5) | Admission  Discharge  Follow-up | 18.05 5.16 4.84 | 3.46 2.61 2.19 | 7.00 2.00 2.00 | 18.00 5.00 5.00 | 22.00 12.00 8.00 |
|  | 3 | 8 (11.7) | Admission  Discharge  Follow-up | 21.38 8.50 0.88 | 4.24 3.70 0.64 | 15.00 3.00 0.00 | 22.00 8.50 1.00 | 28.00 14.00 2.00 |
| DASS-21 Anxiety Total Score | 1 | 51 (63.8) | Admission  Discharge  Follow-up | 11.33 4.71 4.20 | 6.67 5.09 4.65 | 0.00 0.00 0.00 | 10.00 4.00 2.00 | 26.00 24.00 16.00 |
|  | 2 | 24 (36.2) | Admission  Discharge  Follow-up | 27.75 12.33 11.42 | 6.25 5.83 9.86 | 14.00 0.00 0.00 | 28.00 12.00 9.00 | 40.00 24.00 32.00 |
| DASS-21 Stress Total Score | 1 | 13 (18.7) | Admission  Discharge  Follow-up | 20.62 6.00 3.38 | 6.55 3.46 1.26 | 10.00 0.00 0.00 | 20.00 6.00 4.00 | 32.00 10.00 4.00 |
|  | 2 | 27 (34.6) | Admission  Discharge  Follow-up | 29.04 18.00 24.44 | 8.88 6.25 7.49 | 12.00 8.00 12.00 | 32.00 18.00 24.00 | 42.00 30.00 42.00 |
|  | 3 | 35 (46.6) | Admission  Discharge  Follow-up | 28.80 9.54 10.17 | 6.86 4.15 4.84 | 12.00 0.00 0.00 | 30.00 10.00 10.00 | 42.00 22.00 22.00 |
| KPCS Total Score | 1 | 11 (14.7) | Admission  Discharge  Follow-up | 27.45 31.73 31.55 | 5.92 1.95 4.50 | 13.00 29.00 20.00 | 29.00 33.00 32.00 | 36.00 34.00 36.00 |
|  | 2 | 19 (25.3) | Admission  Discharge  Follow-up | 25.42 38.00 38.95 | 2.76 2.38 3.15 | 20.00 33.00 30.00 | 27.00 38.00 39.00 | 29.00 42.00 44.00 |
|  | 3 | 45 (60.0) | Admission  Discharge  Follow-up | 37.31 40.91 40.67 | 3.48 2.08 2.72 | 32.00 35.00 32.00 | 37.00 41.00 41.00 | 44.00 44.00 45.00 |
| MPAS Total Score | 1 | 5 (6.7) | Admission  Discharge  Follow-up | 37.34 51.18 52.46 | 4.65 8.13 4.08 | 33.20 42.30 49.10 | 34.50 49.60 50.10 | 42.70 60.00 58.30 |
|  | 2 | 40 (53.5) | Admission  Discharge  Follow-up | 55.12 72.78 57.63 | 7.13 6.70 4.79 | 37.10 57.90 48.30 | 56.25 75.40 59.20 | 65.60 82.00 65.00 |
|  | 3 | 30 (39.8) | Admission  Discharge  Follow-up | 78.50 81.99 58.57 | 6.32 5.69 4.73 | 64.00 72.90 50.30 | 78.65 81.95 59.15 | 88.60 93.60 67.30 |

EPDS: Edinburgh Postnatal Depression Scale; DASS-21: Depression, Anxiety and Stress Scale-21 item; KPCS: Karitane Parenting Confidence Scale; MPAS: Maternal Postnatal Attachment Scale
